# An Improved Velocity Estimation Method for Wideband Multi-Highlight Target Echoes in Active Sonar Systems

**DOI:** 10.3390/s18092794

**Published:** 2018-08-24

**Authors:** Shuxia Huang, Shiliang Fang, Ning Han

**Affiliations:** Key Laboratory of Underwater Acoustic Signal Processing of Ministry of Education, Southeast University, Nanjing 210096, China; 13404133160@163.com (S.H.); hanning@seu.edu.cn (N.H.)

**Keywords:** multi-highlight echo, wideband ambiguity function, hyperbolic-frequency modulated waveform, Doppler tolerance, sliding window matching

## Abstract

In active sonar systems, the target echoes are usually equivalent to a superposition of the Doppler-scaled reflections from multiple highlights. The reflections overlap with each other both in the time and frequency domain, which results in a decreased velocity estimation performance. Recently, the hyperbolic-frequency modulated signal has been widely employed in sonar systems for moving targets due to its Doppler tolerance, while the precise velocity estimation becomes a great challenge under such conditions. In this paper, the echo c is modeled onsidering a target with a constant velocity and multi-highlights. The velocity estimation performance is analyzed though the signal’s matched filter and the wideband ambiguity function. An improved method based on the sliding window matching algorithm is proposed to improve the performance. The method controls the energy of environmental noise and interference by focusing on the dominant target highlight, and applying a designed window which utilizes the Doppler characteristics of hyperbolic-frequency modulated signals. Simulations and lake experiment allow us to compare between the improved method and the conventional matched filter method. The results verify the influence of the multi-highlights in velocity estimation and indicate that the improved method has more effective performance.

## 1. Introduction

Underwater acoustic target echoes are the physical phenomena which are generated by the interaction of the emission signal, the target and the complicated underwater channel. Some useful information about the target can be extracted from the echo to obtain a series of characteristic parameters [1]. Generally, the complex target echo is regarded as a combination of the reflections from some dispersion highlights [2]. Different reflections have different time-space structures and are aliased in both the time and frequency domains [3]. Therefore, the whole echo can be viewed as equivalent to the summed superposition of the reflections from the multiple highlights, and the respective reflection from each highlight is determined by its spatial distribution and the material of the target [4], also including the information about the velocity, sound intensity and environment conditions, which form the theoretical basis to accomplish the estimation of an underwater target. The target velocity mentioned in this paper totally represents the radial velocity, which is along the line-of-sight of the receiving sonar.

Velocity estimation of underwater targets is a significant element for target recognition and tracking. However, the multiple reflections from multiple highlights overlap severely in the time and frequency domains. Normally, the conventional method estimates the target velocity by computing the center frequency shift in the echo, namely the Doppler shift [5], which is inaccurate for wideband signals. The basic method for active sonar detection system is to apply a linear matched filter (MF) to compute the correlation between the echo and the emitter signal [6]. These signal detection techniques based on cross-correlation methods are used to provide high signal to noise ratio, accurate detection of signal arrival time and good signal recognition from target echoes [7]. Another velocity estimation method is to employ different versions of copied (or replicated) signals with different compression factors in the MF, namely the Doppler-scaled emitter signals [8], to search for the target’s Doppler factor. This method may yield unsatisfactory performance in an actual detection environment due to the multiple reflection points. Very short pulses have come to be used as the emitter signals in some investigations [9,10] and the echo has larger time intervals between the highlights so that the single reflection can be extracted from the time domain. However, the performance will degrade because of the factors such as sound absorption, reverberation and weaker reflection power.

In recent years, researchers have been interested in the applications of the hyperbolic-frequency modulated (HFM) signal, which is maximally insensitive to a constant velocity. The waveform is widely employed in radar and sonar systems for moving target detection [11]. The Doppler tolerance feature of an HFM signal with a large time-bandwidth product will minimize the signal losses [12], because there is also a strong correlation between the echo and a Doppler-mismatched copied signal, which will provide a fantastic detection performance for a moving target [13]. However, the correlation amplitude will deteriorate the velocity estimation performance, especially for multi-highlight targets. The inaccurate velocity will confuse the range estimation in the MF, and such entanglement is termed range-Doppler coupling, which is essentially a compromise for Doppler insensitivity [14]. Although the Doppler tolerance of HFM signals results in poor velocity estimation performance, the parameter is significant and essential for target detection and recognition. Therefore, some improvements should be made since the emitter signal cannot be replaced by Doppler-sensitive signals. The Doppler effect can be properly described as a Doppler compression factor in wideband case [15], so as a result, we investigate the association between the MF output and the main ridge slice of the signal’s wideband ambiguity function (WBAF) in this paper [16]. It can be directly related to the performance of velocity estimation for HFM signals. This study also provides a referential point for emitter signal design.

An improved method of velocity estimation for multi-highlight targets is proposed in this paper. We focus on the reflection point with dominant energy, and then process the partial echo truncated by a sliding window. The starting time and the length of the window are designed based on the inherent feature of HFM signals, and they change with the compression factors in the copied signals. The method provides an effective means for estimating velocity based on processing multiple target highlights, under simulated conditions.

The rest of this paper is organized as follows: Section 2 builds a comprehensive multi-highlight echo model. Section 3 introduces the theory of WBAF and MF, and deduces the ridge amplitude of the WBAF at target velocity. Section 4 provides an improved method to obtain a better velocity estimation performance. Section 5 makes comparisons with the conventional MF method by simulations and underwater experiment, and conclusions are drawn after that.

## 2. An Underwater Echo Model of Multi-Highlight Doppler Targets

**Figure 1:**
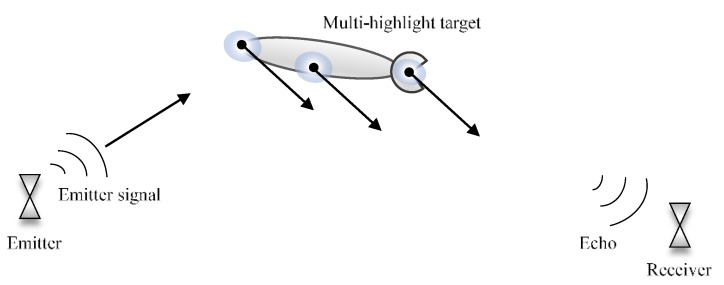
The sonar system and the echo of multi-highlight target.

In the literature, all the echo components can be regarded as the summation of reflections from certain equivalent centers, which is called highlights [17]. Figure 1 depicts the active sonar system and the multi-highlight echo reflected by the target with multiple reflection points.

Compared with the emitter signal, the reflections mainly have several differences in amplitude, time-delay and phase variation of each highlight. The number of highlights and their respective time-delays are mostly determined by the structure of the target. For example, an underwater target can be considered as a finite cylinder, and the target may have several highlights according to geometrical diffraction theory [18].

These highlights are divided into fixed reflection points and moving reflection points: the former have fixed positions on the body of the target, such as the points on the discontinuous edges in the hull structure. The latter are normally the specular reflections on the target surface and move with the target course angle. Taking a submarine as an example, the target can be divided into three equivalent highlights [1]. The echo strength is a function of the emitter signal’s front incident angle relative to the target heading, which determines the orientation distribution of the highlights. 

The complex analytic form with amplitude and phase of wideband sonar signal can be expressed as [1]:

(1) $$u\left(t\right)=u_{c}\left(t\right)e^{j2\pi \varphi \left(t\right)},$$

where φ(t) represents the frequency modulation function of the emitter signal. $u_{c}\left(t\right)=a\left(t\right)e^{j\theta \left(t\right)}$ represents the envelope of the signal, where a(t) is the amplitude function, and θ(t) is the phase modulation function of the envelope. Pulse width broadens or compresses with the target-sonar relative movement, and the echo with a single highlight is expressed as follow [1]:

(2) $$r\left(t\right)=\sqrt{\kappa }u\left(\kappa \left(t-\tau \right)\right)\approx u\left(\kappa \left(t-\tau \right)\right),$$

where κ is the Doppler factor produced by the relative motion between the sonar system and the target, τ is the time-delay represented the respective arrival time of the highlight. In a monostatic sonar system, the theoretical Doppler factor is κ=(c−v)/(c+v), where c denotes the waveform propagation speed, and v denotes the target velocity. We suppose it to be positive when the target moves away from the active sonar system. If c≫v can be satisfied, the amplitude change caused by the target velocity does not need to be considered. Suppose that the initial range of the target is d, the arrival time of the echo is τ=2d/c. The reflection of the single highlight with a constant speed can be expressed as:

(3) $$r\left(t\right)\approx u\left(\frac{c-v}{c+v}\left(t-\frac{2d}{c}\right)\right).$$

The multi-highlight echo is a coherent combination by several reflections from the main highlights on each part of the target [19]. No matter how complex the target is, and the reflection of a highlight can be obtained by the amplitude, time-delay, Doppler factor and phase jump, which is expressed as [1]:

(4) $$r\left(t\right)=\sum_{i=1}^{N}\left\{p_{i}u_{c}\left(\kappa_{i}\left(t-\tau_{i}\right)\right)e^{j2\pi \varphi \left(\kappa_{i}\left(t-\tau_{i}\right)\right)}e^{j\psi_{i}}\right\}+n\left(t\right),$$

where $\tau_{i}=2d_{i}/c$. N is the number of the equivalent highlights, $p_{i}$, $d_{i}$, $\tau_{i}$ and $\kappa_{i}$ compose a set of highlight parameters, that is sound pressure, distance between the sonar, time-delay and Doppler factor. Due to the arrival time when the incident sound wave encounters the highlight, the random phase jump $\psi_{i}$ is introduced in each reflection signal, which is a uniform distributed random variable between [0,2π]. For the smaller reflection point on the curvature area of target, the reflection strength is weaker and not stable. Therefore, this part of reflections will result in fuzzy impulse phenomenon, and they can be regarded as general random noise in simulation. n(t) denotes the sum of these vague reflections and underwater environment noise.

## 3. Wideband Ambiguity Function (WBAF) and Matched Filter (MF) Output

### 3.1. Basic Theory of MF and WBAF

The MF operation between the echo r(t) (as given in Equation (2)) and the copied signal h(t) is written as Equation (5), where the echo includes the time-delay $\tau_{0}$ and the Doppler factor $\kappa_{0}$, that is [6]:

(5) $$\begin{array}{ll} y\left(t\right) & =\int_{-\infty }^{\infty }r\left(\tau \right)\;h\left(t-\tau \right)d\tau =\int_{-\infty }^{\infty }u\left(\kappa_{0}\left(\tau -\tau_{0}\right)\right)\;u^{*}\left(\kappa_{0}\left(\tau -t\right)\right)d\tau \\ & =\int_{-\infty }^{\infty }u\left(\kappa_{0}\tau \right)\;u^{*}\left(\kappa_{0}\left(\tau +\tau_{0}-t\right)\right)d\tau \end{array},$$

where ‘*’ represents the complex conjugate, $h\left(t\right)=u^{*}\left(-\kappa_{0}t\right)$ is the optimal impulse response function of the filter.

Comparing with the MF operation, the WBAF is introduced to perform the estimation of the moving target with the wideband signal. The WBAF is defined as [20]:

(6) $$\begin{array}{ll} \left|\chi \left(\tau ,\kappa \right)\right| & =\left|\int_{-\infty }^{\infty }r\left(t\right)u^{*}\left(\kappa \left(t-\tau \right)\right)dt\right|\\ & =\left|\int_{-\infty }^{\infty }u\left(\kappa_{0}\left(t-\tau_{0}\right)\right)u^{*}\left(\kappa \left(t-\tau \right)\right)dt\right| \end{array},$$

where $\tau_{0}$, $\kappa_{0}$ represent the time-delay and the Doppler factor of the echo, and τ, κ represent the time-delay and the compression factor of the copied signal. According to the relation between target range and time-delay, velocity and Doppler factor, as denoted in Equation (3), Equation (6) can be equivalently written as:

(7) $$\left|\chi_{v}\left(d,v\right)\right|=\left|\int u\left(\frac{c-v_{0}}{c+v_{0}}\left(t-\frac{2d_{0}}{c}\right)\right)u^{*}\left(\frac{c-v}{c+v}\left(t-\frac{2d}{c}\right)\right)dt\right|.$$

Comparing Equation (6) to Equation (5), we can observe the relation between the MF output and the WBAF. From Equations (6) and (7), the WBAF can be seen as the signal energy distribution in the t−κ plane (or the d−v plane). It also describes the similarity between the echo and the copied signals. In other words, the WBAF indicates the velocity estimation result by a two-dimensional image visually. It is a panoramic image of the MF outputs between the echo and the different copied signals. Suppose the target is static (namely $\kappa_{0}=1$), and then the MF output can be written as y(t)=χ(−t,1). It can also be interpreted that the MF output is equivalent to the ‘κ=1’ slice of the WBAF. Obviously, the curve in the ‘κ=1’ slice contains the maximum value of WBAF, namely the peak value of the MF output. For the ‘κ≠1’ slices of the WBAF, the curves do not contain the maximum peak, which is described as ‘mismatch’.

The echo of a moving target is a time-delayed and Doppler-scaled version of the emitter signal. The conventional MF method is to design different copied signals by compressing the emitter signal with different compression factors, and then extract the respective value of the correlation peaks. The compression factor corresponding to the maximum peak is the optimal estimated value, which is approximately equal to the Doppler factor of the target. Then the estimated velocity can be deduced by the optimal compression factor directly.

### 3.2. The WBAF and Doppler Feature of HFM Signal

In order to discuss the characteristics of the multi-highlight echo, the relation between the MF and the WBAF is analyzed from their definitions and outputs. In this paper, the emitter signal is the HFM signal and it is an often-used waveform for target probing [21]. A HFM signal can be defined as follow [21]:

(8) $$u\left(t\right)=A\mathrm{rect}\left(\frac{t}{T}\right)e^{-j2\pi T\frac{f_{H}f_{L}}{f_{H}-f_{L}}\ln\left(1-\frac{f_{H}-f_{L}}{Tf_{H}}t\right)},\;t\in [0,T],$$

where:

(9) $$\mathrm{rect}\left(u\right)=\left\{ \begin{array}{ll} 1, & 0\leq u\leq 1\\ 0, & others \end{array},\right.$$

$j=\sqrt{-1}$, and A is the amplitude that represents the signal energy, rect(t/T) denotes a rectangular envelope, and T is the pulse width. The time derivative of the signal phase is expressed as the instantaneous frequency, f(t), which is given by [21]:

(10) $$f\left(t\right)=\frac{Tf_{H}f_{L}}{Tf_{H}-\left(f_{H}-f_{L}\right)t}.$$

The instantaneous frequency monotonically and continuously goes from $f_{L}$ to $f_{H}$ in a hyperbolic fashion within a pulse duration, and exhibits the time-varying nature of a nonstationary waveform. The bandwidth of u(t) is $B=\left|f_{H}-f_{L}\right|$.

The received target echo includes the time-delay and the Doppler effect. Therefore, let the velocity of a target be v, and κ is the corresponding Doppler factor. Suppose that the initial range of the target is d, namely time-delay is the τ=2d/c. The received HFM signal can be expressed as:

(11) $$r\left(t\right)=A\mathrm{rect}\left(\frac{\kappa }{T}\left(t-\tau \right)\right)e^{-j2\pi T\frac{f_{H}f_{L}}{f_{H}-f_{L}}\ln\left(1-\frac{f_{H}-f_{L}}{Tf_{H}}\kappa (t-\tau )\right)}.$$

The instantaneous frequency of r(t) is $f_{r}\left(t\right)$, as given in Equation (12) [21]:

(12) $$f_{r}\left(t\right)=\frac{Tf_{H}f_{L}\kappa }{Tf_{H}-\left(f_{H}-f_{L}\right)\kappa \left(t-\tau \right)}.$$

If there is a time-delay value $\tau_{1}$ that can satisfy the equation: $f_{r}\left(t\right)=f\left(t-\tau -\tau_{1}\right)$, the signal is the best Doppler insensitive waveform. $\tau_{1}$ can be derived by Equations (10) and (12) [22]:

(13) $$\tau_{1}=\frac{Tf_{H}}{\kappa }\frac{1-\kappa }{f_{H}-f_{L}}.$$

Though the above analysis, a conclusion can be obtained that the HFM waveform minimizes the peak loss of the MF when applied to a moving target with a constant velocity.

**Figure 2:**
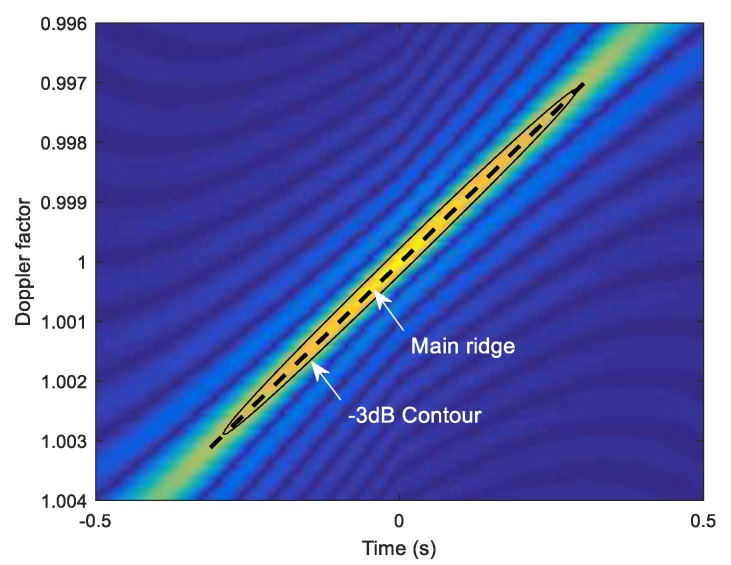
The ambiguity contour plot of HFM signal. The ellipse depicts the ambiguity contour plot, which is the −3 dB contour plot of the WBAF. The dashed line depicts the main ridge of the WBAF.

The WBAF of a HFM signal is shown in Figure 2, where the frequency span is 990~1010 Hz, and the pulse width is 1 s. The analytic expression of the HFM’s WBAF cannot be easily derived, thus the result is generally obtained in numerical method. The maximum value of the HFM’s WBAF locates at the origin of the ambiguity contour plot, and the ambiguity contour plot is an approximate ellipse. The ridge of the WBAF displays on the major axis of the ellipse, and each value on the ridge maps to a MF peak. The decreased peaks imply the Doppler-scaled signals mismatch with the original signal in the MF. Similarly, the velocity estimation for the multi-highlight target can be analyzed through the WBAF ridges of the echo. 

**Figure 3:**
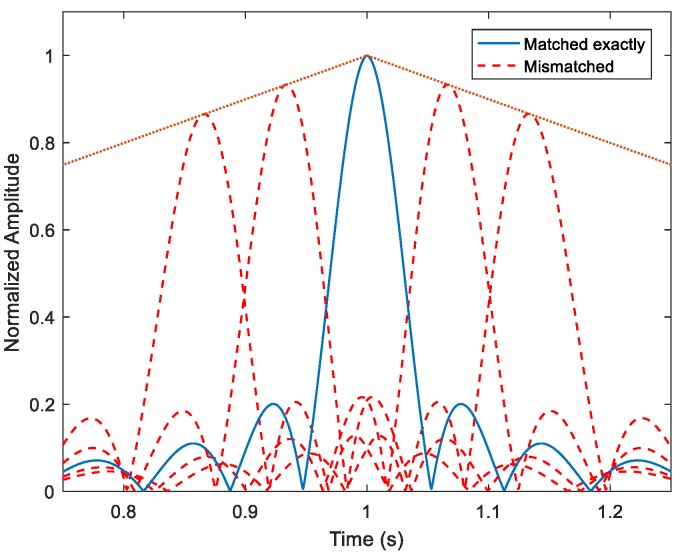
The MF outputs with different Doppler factors. The frequency span is 990~1010 Hz, and the pulse width is 1 s. The dotted line depicts the main ridge slice of the WBAF. The solid line depicts the optimal MF output, and the dashed lines depict the outputs when there are Doppler biases.

In Figure 3, the main ridge of the WBAF is shown as the dotted line. The solid line shows the MF output of the HFM signal when the copied signal has no Doppler bias and exactly matches the original signal. The dashed lines depict the outputs when there are Doppler biases between the copied signals and the HFM signal, and the Doppler bias can be caused by the relative movement of the target. The HFM signal has a prized property of Doppler tolerance, that a waveform which satisfies the property also has a MF output peak for a moving target, even if the time bandwidth product is large [21]. As shown in Figure 3, the output amplitudes of the mismatched copied signals are slightly smaller than the exactly matched (no Doppler bias) copied signal, and they are distributed in sharp peak states as well.

The slope of the WBAF’s ridge can be estimated by the numerical fitting. Moreover, if the instantaneous frequency of the HFM signal approximates a linear function under a limited bandwidth range, its performance can be similar to a LFM signal with the same time and frequency spans. Let P(v) denote the amplitude of the WBAF ridge at velocity v without the consideration of receiver noise, where v=c(1−κ)/2. The power loss ratio (namely the slope of the WBAF ridge) for an HFM signal with rectangular envelope can be loosely approximated as [12,21]:

(14) $$\xi \left(v\right)=\frac{P\left(v\right)}{P\left(0\right)}\approx 1-\frac{2\left|v\right|}{c}\left|\frac{1}{2}-\frac{f_{H}}{f_{H}-f_{L}}\right|=1-\left|v\right|\frac{2f_{0}}{cB},$$

where 0≤ξ(v)≤1, and it is in an approximate linear fashion. The power loss ratio is proportional to the center frequency $f_{0}$, and inversely proportional to the bandwidth propagation speed product Bc. It is related to the estimation performance of target velocity, namely smaller power loss ratio results in degraded velocity estimation performance.

### 3.3. The MF Output and the WBAF of Wideband Multi-Highlight Echo

**Figure 4:**
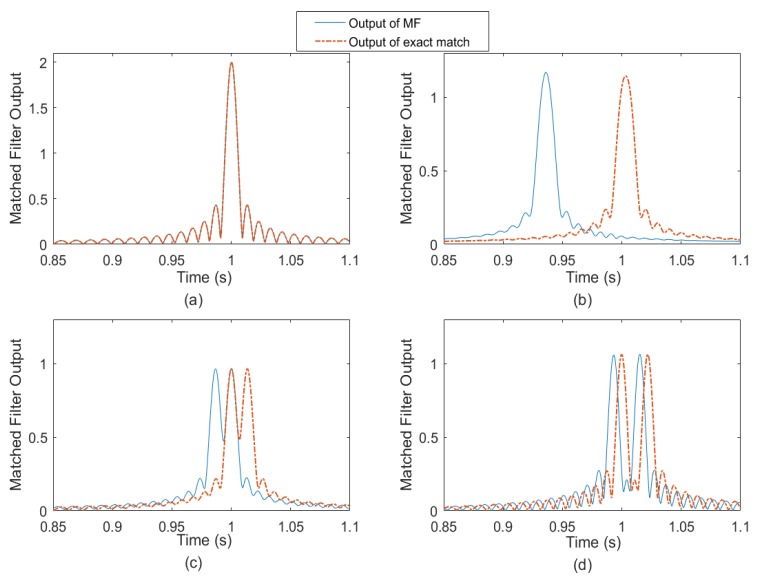
The MF outputs of the two-highlight echoes with different highlight distances L: (**a**) L = 0 m; (**b**) L = 4.5 m; (**c**) L = 10 m; (**d**) L = 16 m. The dash-dotted lines depict the MF output when the echoes and copied signals are matched exactly (no Doppler bias). The solid lines are the outputs from the MF method with its estimated Doppler factors. The frequency span of the emission signal is 990~1010 Hz, and the pulse width is 1 s.

The maximum correlation peak for a single highlight target is relatively simple. However, it can be much more complicated for multi-highlight targets. As stated in Section 2, the echo could be overlapped by several reflections from multi-highlights with different delays. Based on the echo model and the HFM signal mentioned above, we consider a static target including two highlights with the same energy. The target radial size can be seen as the distance of the two highlights [1]. Consider L to be the distance between the two highlights. The arrival time of the first highlight is at 1 s. The MF outputs of the four overlapped echoes are shown in Figure 4.

The dash-dotted lines in Figure 4 depict the MF outputs when the echoes and copied signals are matched exactly (no Doppler bias) in the simulation. The solid lines are the outputs whose correlation peaks become maximum in the MF. As shown in Figure 4, the compression factors corresponding to the maximum peaks may not be the same with the set Doppler factors in simulation. The two reflections of the two highlights completely overlap when L = 0 m, and the echo energy amounts to double, as shown in Figure 4a. Figure 4b shows the MF outputs of the ‘L = 4.5 m’ two-highlight echo. Here the two highlights result in the main lobe broadening, and there is only one peak in the MF output. Moreover, the interaction of the two highlights causes the estimation error of the arrival time. The two main peaks are separated distinctly with the distance L increasing, as shown in Figure 4c,d. The degrading estimation performance of the MF method can be observed apparently, which results from the signal superposition. As shown in Figure 4b–d, the output lines are not coincident. In each figure, the correlation peak value obtained from the copied signal with the set compression factor, is not the maximum. The maximum peak appears in the correlation with a mismatched copied signal. 

According to the theory of correlation, the auto-correlations of the wideband HFM signals have a narrower main lobe than the narrowband signals, which makes them easier to detect [7]. The main lobe width is approximately 2/*B*. However, unlike the narrowband signals, such as the tone signal, the HFM signal also has a sharp cross-correlation peak with a Doppler bias signal. That means it minimizes the signal losses in the case of large time-bandwidth product and high velocity [12,21]. Therefore, in the multi-highlight case, the sharp main lobe and high side lobes of the mismatched correlation can affect the peak values of the other highlights within a short time, and the direct result is that the multiple interactive peak values will confuse the velocity estimation in MF, as shown in Figure 4b–d [14,20].

**Figure 5:**
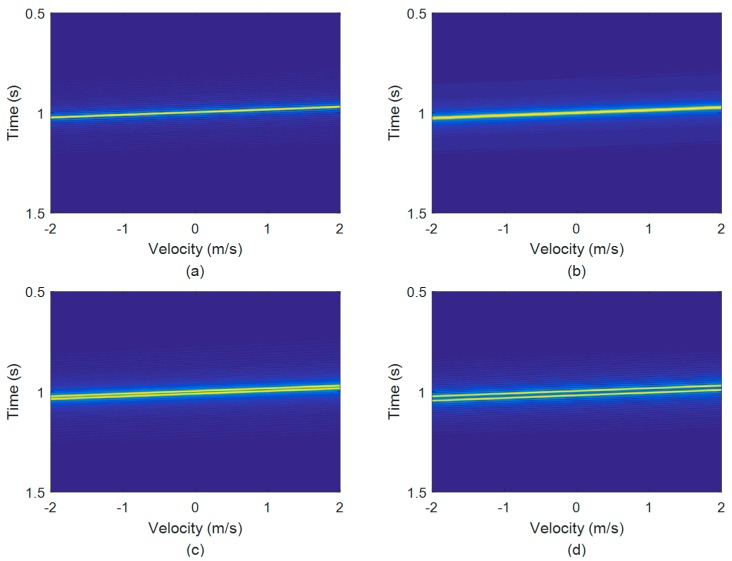
The WBAF outputs of the two-highlight echoes with different highlight distances L: (**a**) L = 0 m; (**b**) L = 4.5 m; (**c**) L = 10 m; (**d**) L = 16 m.

As illustrated in Figure 5, the WBAF outputs of the HFM signals in the same simulation condition are given. The two highlights present a bright main ridge in Figure 5a,b, and present two bright ridges in Figure 5c,d. There is no obviously higher correlation peak in Figure 5, but one or two bright ridges, which results in a poor estimation performance. If the target velocity estimated with a large error, the arrival time of the echo will have difficulty to obtain. Therefore, an effective method is proposed in the next section to improve the performance.

## 4. Velocity Estimation Methods of Multi-Highlight Target with HFM Waveform

### 4.1. The Matched Fitler (MF) Method

For a narrowband signal, that means the bandwidth is much smaller than the center frequency, the target velocity can be estimated by a Doppler frequency shift [23]. However, the usual range of HFM signal bandwidth for target detection and estimation is approximately 50 Hz~1 kHz, and the pulse width is around 0.5~10 s. The frequency shift assumption does not hold because the bandwidth of the signal is comparable to its center frequency.

The MF is a conventional velocity estimation method for wideband signals. It is used for calculating the cross-correlation between the emitter signal and the echo, which is aimed to achieve the maximum signal-to-noise ratio (SNR) of the output [7]. The MF method for velocity estimation makes use of a signal dictionary to match to the echo, which is derived in [24]. The algorithm computes the correlation between the Doppler scaled copied signal and the echo to make a Doppler compensation for the moving target. The steps of the MF velocity estimation algorithm are summarized as follows:

The correlation integral between the received signal r(t) and each dictionary element $h_{m}\left(t\right)\in D$ is computed to obtain the matching filter output, namely substitute Equation (4) into Equation (5). For simplification, we suppose Doppler factor of each target highlight is equal, that is $\kappa_{i}=\kappa_{0}$. The MF output of multi-highlight target is written as:

(15) $$\begin{array}{ll} y_{m}\left(t\right) & =\int_{-\infty }^{+\infty }r\left(t\right)h_{m}^{*}\left(\tau -t\right)dt\\ & \approx \sum_{i=1}^{N}\int_{-\infty }^{+\infty }p_{i}u_{c}\left(\kappa_{0}\left(t-\tau_{i}\right)\right)h_{m}^{*}\left(\tau -t\right)e^{j2\pi \varphi \left(\kappa_{0}\left(t-\tau_{i}\right)\right)}e^{j\psi_{i}}dt \end{array}.$$

In order to best match the target echo with Doppler signal distortions, the copied signals in the dictionary are chosen to be Doppler scaled versions of the emission signal u(t). In this paper, the HFM signal is transmitted, the velocity parameter sampling rate is chosen as $\delta_{v}$, and the expected velocities are bounded in v∈[−V,V]. Then, the copied signals in the dictionary D are obtained as:

(16) $$h_{m}\left(t\right)=e^{-j2\pi T\frac{f_{H}f_{L}}{f_{H}-f_{L}}\ln\left(1-\frac{\left(f_{H}-f_{L}\right)\kappa_{m}t}{Tf_{H}}\right)},\;m\in \left[1,2V/\delta_{v}+1\right],$$

where $f_{L}$, $f_{H}$ and T in the copied signals are the same as the parameters of the emitter signal. The compression factor of the *m*-th copied signal is obtained by:

(17) $$\kappa_{m}=1-\frac{2}{c}\left(-V+\left(m-1\right)\delta_{m}\right).$$

Extract the correlation peaks of the MF outputs generated in Equation (15) to compose the peak value set $ℜ=\left\{M_{1},\ldots ,M_{m},\ldots ,M_{2V/\delta_{v}+1}\right\}$, where $M_{m}=\;\max\left|y_{m}\left(t\right)\right|$. Then the optimal copied signal from the dictionary is the one that maximizes the amplitude of the matching output:

(18) $$h_{opt}\left(t\right)=\underset{h_{m}\in D}{\arg\max}\left\{M_{m}\right\}.$$

The corresponding optimal factor $\kappa_{opt}$, which approaches to $\kappa_{0}$, can be obtained through the comparison among the peak values in ℜ. Meanwhile, the estimated target velocity is expressed in Equation (19):

(19) $$\hat{v}=\frac{c}{2}\left(1-\kappa_{opt}\right).$$

An example is given to show the performance of the MF method. Let a HFM waveform be the emitter signal, where the frequency span is 300–400 Hz, the pulse width is 4 *s*. The target includes three highlights, that the energy ratio is 1:1:0.3. The radial distance between them are both 30 m (suppose the three highlights are in a straight line). The velocity of the target is 3.5 m/s. The parameters and conditions of the simulations below will not change without a specific instruction.

**Figure 6:**
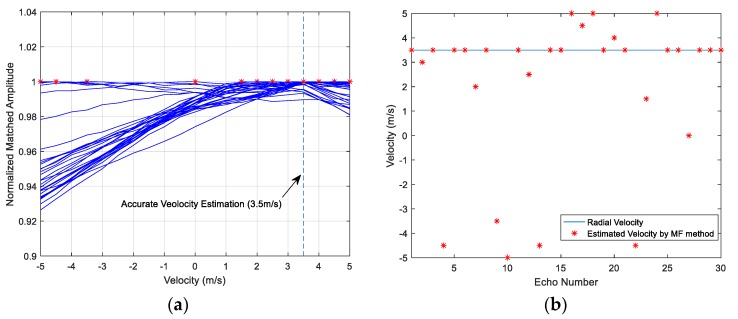
(**a**) Peak value lines of 30 simulations; (**b**) The target velocity estimation result of the MF method.

Thirty simulation results are illustrated in Figure 6a by 30 lines. Gaussian noise and the random phase shifts of the multi-highlight reflections are changed in the 30 simulations. The horizontal axis denotes the velocity value which corresponds to the compression factor in the copied signal. The vertical axis denotes the amplitude of MF output. Each line in Figure 6a links the MF peak values of different copied signals from the dictionary, namely the line links the elements in the peak value set ℜ in a simulation. Each maximum peak value, which is plotted in asterisk ‘*’, indicates the final estimated velocity in this simulation. As shown in Figure 6a, some peak value lines do not have apparent tilt, as a result, their maximum peak values do not converge, and may become outliers deviating from the target truth value.

Figure 6b indicates the respective estimation result of the 30 simulations. The horizontal axis denotes the simulated echo number (1~30), and the vertical axis denotes the estimated velocity. The solid line indicates the set radial velocity 3.5 m/s, and each asterisk indicates the result of the corresponding simulation. The statistical results are unsatisfactory for the MF method as they indicate an accuracy of less than 50 percent.

The MF method cannot obtain ideal velocity estimation from the multi-highlight echo due to the waveform overlap. Because of the Doppler tolerance of the HFM signal, the MF output amplitude does not decline sharply when the target has a relative velocity. For the reason, the envelope side lobe of each highlight will affect the peak amplitude of the other highlights. However, the maximum peak value is the significant basis to estimate the target velocity, thus we often obtain erroneous estimation from the complicated echo of a multi-highlight target.

The estimation performance of the multi-highlight target, which has significant differences between the energy of the highlights, may maintain the same performance as the target with only one reflection point. However, if the energy of the highlights is not much different from each other, the estimation accuracy cannot be accepted. One reason is that the peak value may be obtained by different highlights, namely which highlight contributes to the maximum peak value cannot be distinguished, so that the maximum value cannot be the reliable basis to estimate the target velocity any more, especially in the situation of random phase shift and underwater noise. Therefore, the conventional method needs some improvements.

### 4.2. Focusing on the Dominant Highlight

The main reason influencing the estimation performance is the signal superimposition caused by the multi-highlights of the target. In this section, the problem about ‘All of the highlights with similar energy could determine the maximum peak value’, is solved by focusing on a dominant highlight. The detailed process is to confirm a dominant highlight by comparing the peak values of MF output, and then observing the considered highlight. For example, as shown in Figure 4c,d, the multi-highlight echoes have two peaks in the MF output, respectively. The improvement is to choose the first highlight to be the dominant, namely the first peak value is added into the comparison, rather than the absolute maximum of the whole MF output.

Generally, the first arrival component of the multi-highlight echo is one part with high energy because of the shorter propagation distance and less energy loss in the underwater channel. The component has a clear part in the front of the reflection and only overlaps in the latter part. As a result, the simulation in this paper supposes the first highlight has the dominant power and its correlation peak value is the basis to estimate the target velocity. However, in actual underwater environment, the first arrived highlight may not have the greatest reflection energy. For example, a specular reflection may occur on the bridge section of a submarine, which concentrates more energy [1]. Therefore, the dominant highlight should be selected carefully, by considering the echo structure in the actual applications.

**Figure 7:**
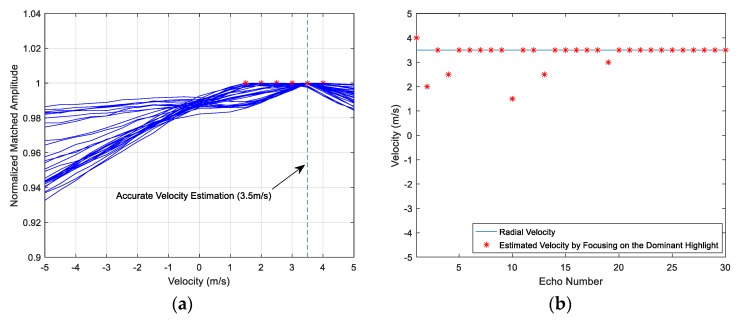
(**a**) Peak value lines of 30 simulations; (**b**) The target velocity estimation result of the process that focusing on the dominant highlight.

A recommended process to select a highlight is that if the first few correlation peaks have high amplitudes, then the first highlight can be focused on; otherwise, the highlight which has the obviously dominant energy can be taken into comparison. According to the Doppler tolerance of HFM signal mentioned above, there will be a peak at the MF output whether the copied signal and echo are matched exactly or not [21], as shown in Figure 4c,d. Therefore, by computing the correlation between the echo r(t) and the emitter signal u(t), the dominant highlight can be considered. Then the peak value set in Equation (18) is composed by the MF amplitudes of the dominant highlight under different compression factors. The process makes some progress of the performance, as shown in Figure 7a,b, the proposed improvement could effectively eliminate outliers.

As depicted in Figure 7, the accuracy of estimated target velocity is improved by focusing on the dominant highlight; however, the estimation performance is still unsatisfactory after the initial optimization and a variable-length sliding window matching method is proposed in Section 4.3 to improve the estimation further.

### 4.3. The Improved Method

On the basis of the improvement mentioned above, a time-window is applied to reduce the energy proportion of the other disturbing reflections. It is proved that if the window contains the complete length of the reflection signal, the energy of the signal can be retained and the absolute value of the MF output maximizes at the arrival time of the impulse [25]. The time coordinate of the peak in MF output is the arrival time of the corresponding highlight when the emission signal matches to the echo exactly (no Doppler bias). Based on the Doppler characteristic of HFM signal, the time coordinate does not equal to the true arrival time for v≠0, and a certain amount of time bias exist [21], as derived in Equations (12) and (13). 

Inspired by the thought of signal processing in multipath channels [24,25], we propose an improved method based on the MF algorithm and the sliding window. The improved method for multi-highlight targets is to focus on the dominant highlight and utilize the sliding window matching technique. The starting point and the length of the sliding window are designed according to the Doppler tolerance of HFM waveform.

The procedures of the improved method to estimate target velocity are summarized as follows:

*Step* *1.* Compute the correlation between the echo r(t) and the emitter signal u(t) to consider the dominant highlight. Then extract the arrival time $t_{0}$ of the dominant highlight from the MF output.
*Step* *2.* Build the complete dictionary of the copied signals, as introduced in Section 4.1.
*Step* *3.* Compute the time coordinate bias of the dominant highlight, the formula is given as follows:
  (20) $$\tau_{i}=\frac{1-\kappa_{i}}{\kappa_{i}}\frac{Tf_{H}}{f_{H}-f_{L}}\approx \frac{2v_{i}Tf_{H}}{c\left(f_{H}-f_{L}\right)},\;i\in \left[1,2V/\delta_{v}+1\right].$$
  Equation (20) is derived from Equation (13), which is a deduced theoretical conclusion of HFM signal.
  Cut off the echo by a time window, where the window length is $T/\kappa_{i}$, the starting time of the window is:
  (21) $$t_{i}^{start}=t_{0}-\tau_{i}=t_{0}-\frac{2v_{i}}{c}\frac{Tf_{H}}{f_{H}-f_{L}}.$$
  Set the sampling values outside the window as zero, and then a new echo $r\prime_{i}\left(t\right)$ is constructed:
  (22) $$r\prime_{i}\left(t\right)=\left\{ \begin{array}{ll} r\left(t\right), & t\in \left[t_{i}^{start},t_{i}^{start}+\tau /\kappa_{i}\right]\\ 0, & others \end{array}\right..$$
  Scan the dictionary and compute the MF outputs $2V/\delta_{v}+1$ times to prepare for the estimation in the next step.
*Step* *4.* Extract the correlation peak values of the MF outputs generated in Step 3 to compose the peak value set $ℜ=\left\{M_{1},\ldots ,M_{m},\ldots ,M_{2V/\delta_{v}+1}\right\}$. The estimated velocity can be obtained through the comparison among the peak values in ℜ, as expressed in Equations (18) and (19).

**Figure 8:**
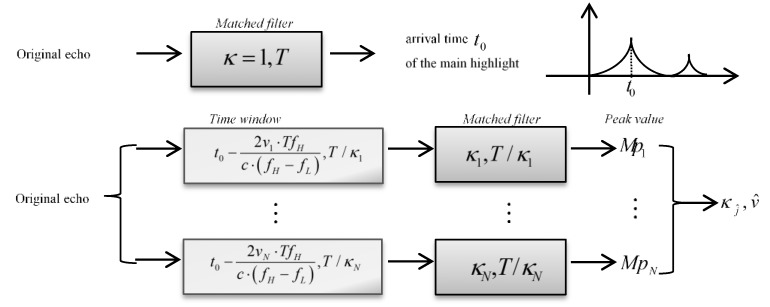
The block diagram of the improved method of the target velocity estimation (The two parameters in the Matched filter block represent the Doppler factor of the copied signal and the pulse duration. The two parameters in the Time window block denote the starting point and the length of the window respectively).

The block diagram of the procedure is depicted as Figure 8, where the parameters in the ‘Matched filter’ block represents the compression factor and the pulse duration of the copied signal respectively. The two parameters in the ‘Time window’ block denote the starting point and the length of the window respectively, which is described as Equations (21) and (22). Firstly, compute the correlation between the original echo and the emission signal (whose compression factor and pulse duration is 1 and T), to consider the dominant highlight. Then, let the echo through a time window, and compute the MF outputs with the corresponding copied signals. The compression factor and the pulse duration of the copied signal is $\kappa_{i}$ and $T/\kappa_{i}$, where $i\in \left[1,N\right],\;N=2V/\delta_{v}+1$. Repeat the operation N times, and obtain the estimated Doppler factor after the comparison among the MF peak values. Finally, the estimated velocity can be derived from the estimated Doppler factor.

The improved method is equivalently to change the upper and lower bounds of the integral formula in the MF processing. Through the window matching algorithm, the MF output is the correlation between the truncated echo and the copied signal. It is certainly less than the whole echo, whereas the contribution of the dominant highlight in the echo is greater. In other words, the energy proportion of the dominant highlight to the total echo increases a lot.

The purpose of the time window is to ensure the signal component of the main highlight is more than the others in the reconstructed echo, so that the integral length of other highlights is limited, the secondary peaks and their side lobes of the MF output are restrained. The key technology of the window design is the starting point and the window length, Step 3 provides the reliable basis for the signal processing in Step 4. According to the Doppler tolerance of HFM signal, there is a nearest arrival time from the designed starting points in Equation (21), and the corresponding window contains the most complete component of the dominant highlight. Furthermore, the other components are truncated and their proportion of the processing signal are decreased.

**Figure 9:**
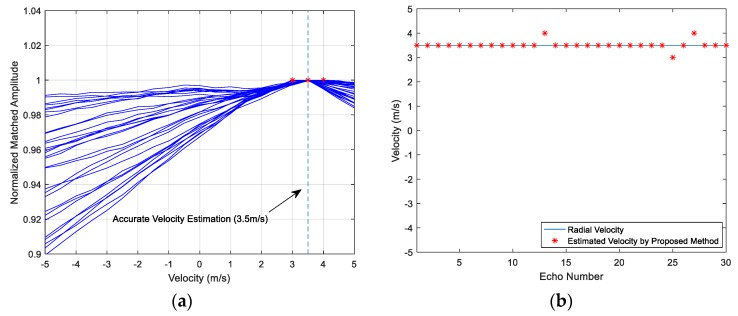
(**a**) Peak value lines of 30 simulations; (**b**) The target velocity estimation result of the final improved method.

The method can be simply divided into two parts. The first part is to identify and select the dominant highlight of the reflection points, and then focus on it. The next part is to apply a sliding window to realize the segment matching operation. The velocity estimation results of the improved method are illustrated in Figure 9a,b. Each line in Figure 9a denotes a simulation that only generates new environment noise and random phase shift of each highlight. The declines of the peak value line become steeper than the lines in Figure 6a and Figure 7a. The estimation velocities converge to a reasonable range [3 m/s, 4 m/s]. Clearly, the accuracy of the improved method increases a lot and the target velocity can be estimated precisely almost every time in this condition.

The proposed method is a progressive optimization technique, and it is based on the conventional MF method. The simulation results are shown in Figure 6, Figure 7, and Figure 9, and the estimation performance is improved step by step. Compared to the MF method, the proposed method does not compute the correlations with the echo directly. It considers the influence of the multi-highlights and provides an improved algorithm to handle the difficulty. The method brings better performance by focusing on the dominant highlight and applying the variable-length sliding window to match the echo, and it makes full uses of HFM’s Doppler tolerance to design the time window. The method controls the energy proportion of noise and other interference in the multi-highlight situation to achieve a satisfactory velocity estimation. 

## 5. Simulations and Underwater Application

In this section, the previously described methods in Section 4.1 and Section 4.3 are tested on three sets of parameters in simulations, and a dedicated real data set recorded in a shallow water environment. The results are analyzed to compare their performance of velocity estimation. 

### 5.1. The Simulations of the MF and Improved Methods

In this section, two methods of target velocity estimation are compared in simulations, namely the MF method, the process that focusing on the dominant highlight and the improved method for wideband multi-highlight signal. Three examples are presented to verify the improved method, and three parameter sets are used:

- Case 1: {300 Hz≤f≤400 Hz, T=4 s, SNR=5 dB, v=3.5 m/s};
- Case 2: {300 Hz≤f≤400 Hz, T=4 s, SNR=0 dB, v=3.5 m/s};
- Case 3: {300 Hz≤f≤500 Hz, T=8 s, SNR=5 dB, v=3.5 m/s}.

**Figure 10:**
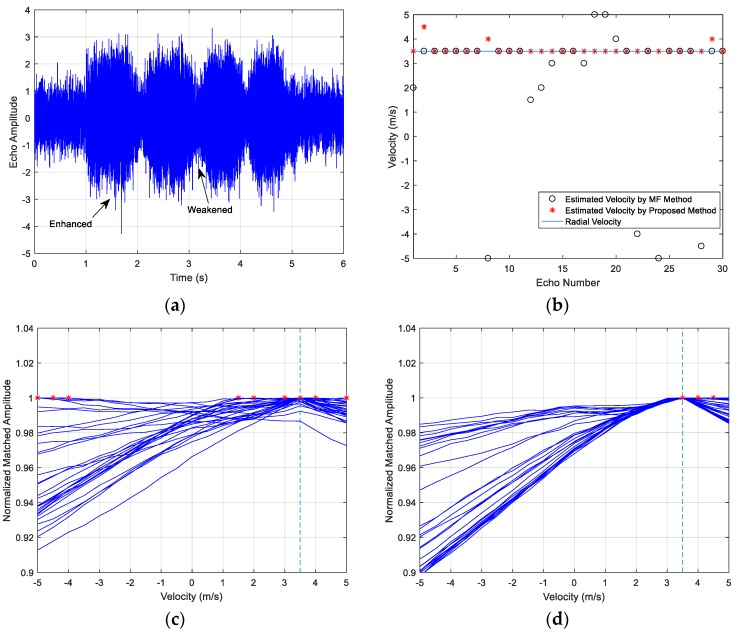
Case 1 results: (**a**) The target echo of the signal; (**b**) The target velocity estimation results of the two methods; (**c**) 30 Peak value lines of the MF method; (**d**) 30 Peak value lines of the improved method.

**Figure 11:**
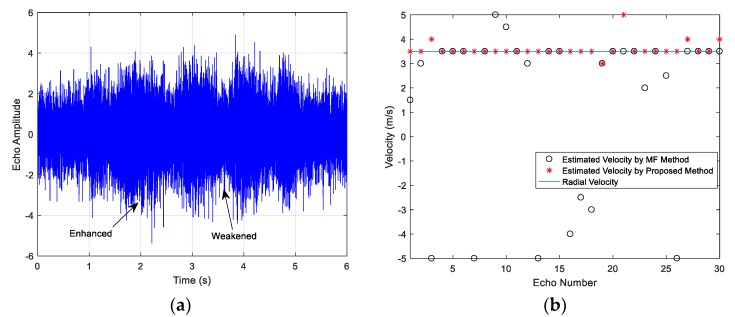

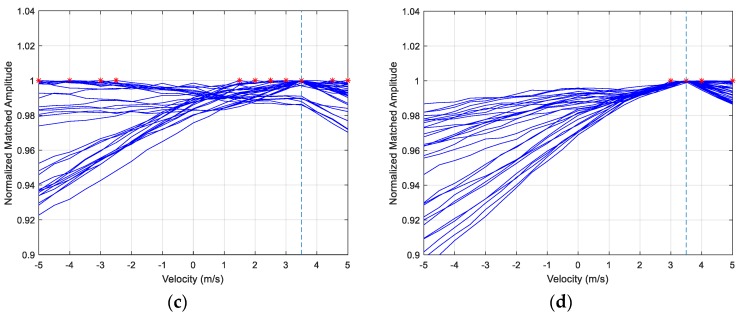
Case 2 results: (**a**) The target echo of the signal; (**b**) The target velocity estimation results of the two methods; (**c**) 30 Peak value lines of the MF method; (**d**) 30 Peak value lines of the improved method.

**Figure 12:**
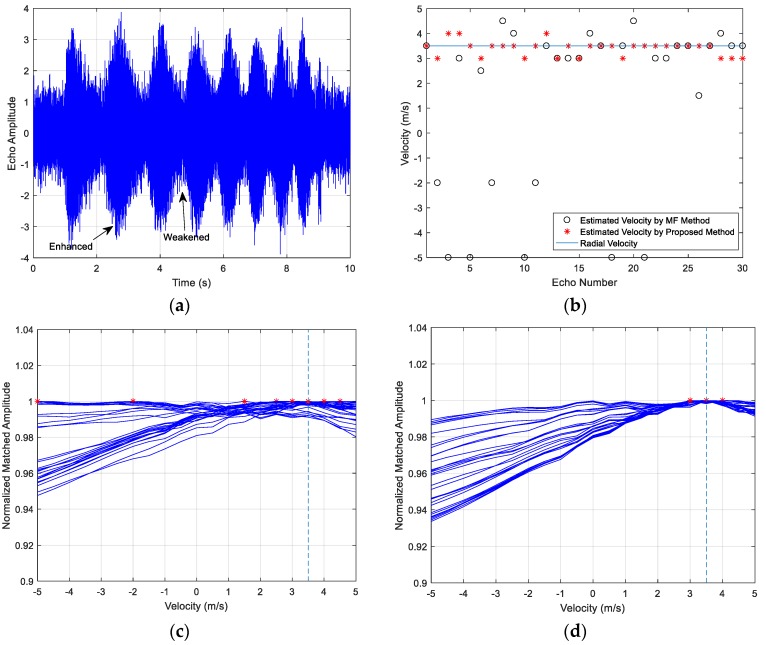
Case 3 results: (**a**) The target echo of the signal; (**b**) The target velocity estimation results of the two methods; (**c**) 30 Peak value lines of the MF method; (**d**) 30 Peak value lines of the improved method.

The emitter signals in the three examples are HFM waveforms and the signal parameters include the frequency range, the pulse width, the SNR and the target velocity. The examples share the same target with a constant speed. The target contains three highlights and the amplitude ratio is 1:1:0.3, the respective separation distance is 30 m. The acoustic propagation speed is c=1500 m/s. The time-domain echoes of the three examples are shown as (a) in Figure 10, Figure 11 and Figure 12, where the respective echo arrival time of the three cases is at 1 s in simulations. Figure 10b, Figure 11b and Figure 12b represent the velocity estimation results of the MF method and the improved method by focusing and sliding window matching, where the solid line represents the set radial velocity, the asterisk ‘*’ denotes the estimation results of the improved method and the black circle ‘o’ denotes the estimated velocity of MF method. Figure 10c,d, Figure 11c,d and Figure 12c,d respectively illustrate thirty MF peak value lines of the two methods in each simulation case and the maximum peak values are marked. From these figures, we observe the following:

- The amplitude of echo is enhanced or weakened at some time, as shown in Figure 10a, Figure 11a and Figure 12a, and the fluctuation is caused by the overlapped reflections from multi-highlights.
- The estimated velocity values distribute on both sides of the actual radial value. Many estimations of the MF method are outliers, and the results have the biggest estimation errors in the three cases. The accuracy of the proposed method results is improved obviously and it provides a more accurate estimation.
- The estimation accuracy decreases along with the SNR dropping, as shown in the comparison between Case 1 and Case 2, and the target reflection impulses become fuzzy in the received waveforms.
- The estimation accuracy of Case 3 is slightly worse than Case 1, since the power loss ratio of the Case 3 signal’s WBAF is smaller than the Case 1, which can be deduced in Equation (14). The power loss ratio of WBAF is related to $f_{0}/B$ of the signal. The ratio of Case 1 is 3.5, and the ratio of Case 3 is 2. The parameters of the Case 3 signal influence its velocity estimation performance, namely smaller power loss ratio results in worse velocity estimation performance under multi-highlight conditions.

The specific statistical results of the velocity estimation are shown in Table 1, and the performances of the two methods can be observed from the mean squared error (MSE) of the velocity estimation. The velocity estimation MSE represents the accuracy and stability of the estimation method, and it can be obtained as follows:

(23) $$MSE_{v}=\sum_{i=1}^{N}\frac{{\left({\hat{v}}_{i}-v_{0}\right)}^{2}}{N},$$

where N is the number of simulations, $v_{0}$ is the set value and ${\hat{v}}_{i}$ is the estimated velocity in the i-th simulation.

The velocity estimation MSE of the two methods can be compared by the last two columns in Table 1. Case 1 and Case 2 share the same parameters and format of the emitter signal, while the SNR of Case 2 is 0 dB, which is lower than Case 1. Simulation results show that the estimation accuracy of Case 2 is worse than Case 1. The center frequency bandwidth ratio $f_{0}/B$ in Case 1 and Case 3 are 3.5 and 2 respectively, which indicates that the power loss ratio of WBAF in Case 3 is smaller than Case 1, therefore the estimation accuracy of Case 3 is worse than Case 1. The improved method provides a higher velocity estimation accuracy in different situations, and its result is more stable, which demonstrates the superiority and robustness of this method.

### 5.2. Application to Underwater Acoustic Data from the Active Sonar 2017 Lake Experiment

The Active Sonar 2017 Lake experiment was conducted by the Key Laboratory of Underwater Acoustic Signal Processing of the Ministry of Education at Southeast University. The real data was collected from a shallow water environment of a lake in Zhejiang Province, China. A HFM signal was emitted by an active sonar system and the echo was reflected from a moving target at slowly varying velocity (7~10 m/s) and with a sonar-target separation ranging from 700 to 3000 m. The emitter HFM signal had a central frequency of 1625 Hz with a 500 Hz bandwidth, and a duration of 2 s. 

**Figure 13:**
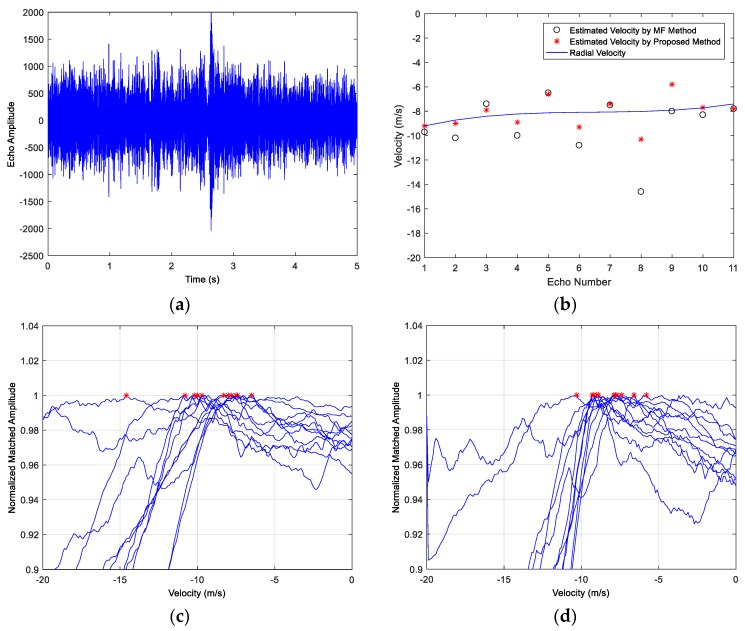
Active Sonar 2017 Lake Experiment results: (**a**) The first target echo; (**b**) The target velocity estimation results of the two methods; (**c**) 11 Peak value lines of the MF method; (**d**) 11 Peak value lines of the improved method.

Eleven echoes were collected and processed by the MF method and the improved method in this paper. Figure 13a is the time-domain waveform of the first echo, and the reflection pulse is almost indistinguishable from the received signal. Figure 13b illustrates the velocity estimation results, where the solid line links the approximate radial velocities, the asterisk ‘*’ and the black circle ‘o’ denote the estimation results of the improved method and MF method respectively. The real velocity in this processing varies from −9 to −8 m/s (the target moved towards the sonar system). The deviations from the radial velocities become smaller by the improved method, namely the results of the convenient MF method have a bigger MSE of the estimation. The proposed method utilizes a variable-length sliding window matching algorithm to increase the energy proportion of the dominant highlight. As shown in Figure 13c,d, the maximum peak values (corresponding to the estimated velocities), which are plotted in asterisks ‘*’ are converging. The slopes of the peak value lines in (d) are bigger than (c), therefore, the maximum peak values become more easily to figure out, and the estimation accuracy increases by the improved method. It can be noticed that the result accuracy of the improved method does not reach the accuracy in simulations, and the estimation error may come to 1 m/s. This is most likely due to the factors of the multipath and the low SNR of the real environment. However, the estimation results of the real data indicate that the improved method proposed in this paper has a better velocity estimation performance in actual application.

## 6. Conclusions

In this paper, a complete multi-highlight echo model is built, including the information of target multi-highlights, velocity, and simulated underwater conditions. Due to the large application range of HFM signals and the inevitable requirement for target characteristics, we have to improve the conventional MF target velocity estimation method to obtain a better performance. The Doppler insensitivity of the HFM signal is investigated through analyzing the MF output and the WBAF. The internal factors of the poor performance are the design parameters of HFM waveform, over which the design agent has full control (i.e., formatting the emitter signal), while the external factors are the parameters of the target and environment (i.e., echo overlap caused by multi-highlight target, underwater channel), over which the design agent has limited control. To achieve accurate velocity estimation, an effective method based on the technique of focusing on the dominant highlight and the algorithm of variable-length sliding window matching is proposed according to these theoretical achievements. It makes full use of HFM’s Doppler tolerance to design the time window. The improved method provides better performance by controlling the energy proportion of noise and other interference in a multi-highlight situation. The realizability and stability of the method are confirmed by simulations, and the comparisons between the improved method and the MF method are shown by figures and statistical data. The improved method was also tested on real set of data from 2017 Lake Experiment leading to realistic results. It indicates an improved estimation performance, although the result accuracy does not reach the accuracy in simulations. The possible reasons may be the multipath and low SNR of the real underwater environment and our future work will focus on dealing with these problems.

## 7. Patents

Fang S.; Huang S. A Velocity Estimation Method Based on Multi-highlight Target Model. China Patent 201610552899.7, 14 July 2016.

## Figures and Tables

**Table 1:** The mean squared error analysis of the MF method and the improved method.

Frequency Range (Hz)	Pulse Duration (s)	SNR (dB)	Velocity Estimation MSE	
			MF Method	Method of Focusing and Sliding Matching
300~400	4	5	9.2833	0.0148
300~400	4	0	14.492	0.1762
300~500	8	5	15.375	0.1809
